# Virtual Reality as a Digital Premedication to Alleviate Preoperative Anxiety and Postoperative Pain in Patients Undergoing Spine Surgery: Study Protocol for a Randomized Clinical Trial

**DOI:** 10.3390/brainsci16060587

**Published:** 2026-05-29

**Authors:** Redwan Jabbar, Samuel D. Pettersson, Agnieszka Pawelczyk, Maciej Radek

**Affiliations:** 1 Department of Neurosurgery, Spine and Peripheral Nerves Surgery, Medical University of Lodz, 90-549 Lodz, Poland; 2 Department of Neurosurgery, Medical University of Gdansk, 80-210 Gdansk, Poland; 3 Neurosurgical Service, Beth Israel Deaconess Medical Centre, Harvard Medical School, Boston, MA 02115, USA

**Keywords:** preoperative anxiety, postoperative pain, virtual reality, VR, randomized clinical trial, RCT

## Abstract

**Background**: Preoperative anxiety and postoperative pain are prevalent and are frequently associated with poor postoperative functional outcomes. Comprehensive postoperative management, including both pharmacological and psychological components, is essential for proper postoperative care and better recovery. While the analgesic effect of traditional non-pharmacological intervention, such as cognitive behavior therapy, has been investigated by other trial studies, the newer innovative methods for delivering psychological interventions for reducing anxiety and pain are extensively being investigated. Virtual reality (VR) has emerged as a novel and promising technology that offers opportunities to mitigate patient perception and cognitive responses, and has been shown to be associated with lower levels of anxiety and pain. The aim of this randomized clinical trial (RCT) is to determine whether delivering the psychological content through virtual reality (VR) along with the standard preoperative and postoperative care results in better anxiety and pain relief outcomes than standard care in patients undergoing spinal surgery. **Methods**: This study protocol outlines a parallel-group RCT to be conducted in the Department of Neurosurgery at the University Clinical Hospital of Medical University of Lodz. The objective is to assess the efficacy of immersive VR environments in reducing preoperative anxiety and postoperative pain intensity in the following day after surgery. Adult patients (18–70) will be randomly assigned to either (1) standard care before surgery (control group), (2) VR exposure simulating the hospital environment alongside standard care, or (3) VR-based exposure to calming natural landscapes accompanied by soothing background sound along with standard care. In each group, a minimum of 50 patients will be recruited. The primary outcome is the change in preoperative anxiety measured using the State-Trait Anxiety Inventory-State (STAI-S) scale from baseline to immediately after intervention. Secondary outcomes include postoperative pain measured using the Visual Analogue Scale (VAS), postoperative analgesic consumption, patient satisfaction, and VR-related adverse effects. To facilitate a comprehensive understanding of the VR intervention’s impact, the primary outcome will be complemented with measures of the adverse effects, level of immersion, and level of presence in the VR environment. Secondary outcomes of self-reported satisfaction scores and postoperative analgesics from patients’ medical charts will be assessed. **Conclusions**: This trial will evaluate whether VR-based interventions may reduce preoperative anxiety and postoperative pain in patients undergoing spine surgery. This study may provide evidence supporting the future implementation of VR as a non-pharmacological adjunct in perioperative care. This intervention may hold significant clinical relevance clinically, particularly in patients with high level of preoperative anxiety, by offering an alternative method to pharmacological anxiolytics in the future.

## 1. Background

Preoperative anxiety and stress are a frequently encountered phenomenon among patients, while such anxiety is experienced on different levels based on sociodemographic characteristics. The overall prevalence of preoperative anxiety varies and is reported in a range of 60–80% while other studies showed a wider range of 11–80% [1,2,3,4,5]. It occurs as a compatible and physiological response to surgical encounters and stress, which might occur anytime preoperatively [6]. The anxiety and stress are induced during hospitalization and intensified when a patient is informed about the need to undergo surgical intervention, and shortly before surgery [7].

Preoperative stress-related signs are associated with a potential change in patients’ psychological responses and hemodynamic parameters, including increased blood pressure and heart rate, that may pose a danger to the patients’ health [8]. Thus, these changes might affect the quality of anesthesia and demand for analgesics, postoperative pain, and possible delirium [9].

It also has significant impact on recovery, including longer postoperative hospital stay and even cognitive and behavioral ramifications. Thus, procedures which are painful lead to preoperative and postoperative anxiety, which causes fear in patients. This leads to a reduced pain threshold and higher postoperative pain and can affect patients’ compliance with subsequent future procedures [10]. A meta-analysis revealed that social support is crucial in reducing the postoperative anxiety among patients undergoing elective surgery. Conventional methods and interventions for managing pain and anxiety typically involve patient education and therapy with anti-anxiety medications prior to surgery to enhance patients’ comfort. However, randomized controlled trials have reported that these approaches provide only limited effectiveness and are associated with risks such as side effects, dependence, drug tolerance, and prolonged recovery time. As a result, there is increasing demand for non-pharmacological interventions to manage pain and anxiety, mitigating the drawbacks and risks associated with this traditional approach [11,12].

Research studies have shown that addressing pain through non-pharmacological intervention, such as cognitive behavioral therapy, music therapy, and interacting with immersive VR, results in a slower response to incoming pain signals [13]. Pain distractions such as television, music and games have been shown to have less of an effect on pain reduction than immersive VR exposure. Therefore, controlled preoperative anxiety and decreased pain reduce hospital stay lengths, healthcare costs, and postoperative complications. Pharmacological intervention is a current method used to reduce anxiety and pain preoperatively; however, it has been associated with a variety of risks which patients are facing, such as addiction, anaphylaxis, polypharmacy, and tolerance development [14]. The integration of non-pharmacological approaches has been one of the main priorities of healthcare reform in the last few years.

Current practice guidelines recommend that non-pharmacological interventions such as distraction and procedural preparation are routinely implemented in treatment plans. Distractions have been reported to have scientific validity as an effective non-pharmacologic intervention to reduce pain and anxiety in both pediatric and adult patients in clinical settings [15]. VR is considered to be an alternative type of non-pharmacologic anesthesia to modulate pain which helps with drawing attention and decreasing the amount of pain perceived [16]. Over the last few years, recent studies have assessed whether VR is suitable for the delivery of enhanced distractions and patient preoperative preparation interventions.

Studies have found that the decrease in pain experienced by the VR group is significantly less compared to the control group. Two systematic reviews have supported these findings; they studied the impact of VR on ninety-eight patients with hand injury undergoing dressing change.

Guo et al. (2015) [17] researched the effectiveness of virtual-reality-based distractions on pain among a group of patients with hand injuries during their dressing change. Ninety-eight patients were part of the control group and intervention group; the former scored better after the dressing change, and a statistical correlation between involvement and engagement in VR was found to significantly decrease pain.

Schwartz et al. (2020) [18] conducted a quality improvement VR project to not only reduce pain but also perioperative anxiety in pediatric burn patients. Forty-six children were given either VR or another distraction before undergoing their dressing change. Post-treatment scores were statistically more effective in the first group, with VR outperforming in each category.

Over the past five years, most of the research surrounding VR found it to be beneficial in clinical and surgical settings. Dehghan et al. (2019) researched the benefits of virtual reality on preoperative anxiety in children, where 40 children were allocated into two groups (control and intervention) [9]. The results indicated considerable changes for the intervention group from baseline to post-test, demonstrating that VR can substantially reduce anxiety in children in the preoperative setting. Sweta et al. (2019) studied the use of VR on patients following a dose of local anesthesia, gathering statistically significant improvements for preoperative and postoperative hemodynamic parameters and on the postoperative visual analog pain scale [19]. Findings from Moon et al. (2018) also highlighted how VR outperforms midazolam on patients’ and anesthesiologists’ satisfaction scores [20]. Therefore, three independent and systematic reviews have concluded that VR is effective at reducing acute and procedural pain through means of a distraction mechanism and may help to create an anxiolytic effect [21,22].

Therefore, the summarized findings suggest that immersive VR is a feasible and an alternative novel non-pharmacological method for reducing preoperative anxiety, improving and optimizing pain management. The results support its application and highlight its potential to enhance clinical outcomes across a variety of clinical settings.

Studies have reported that patients using anxiety reduction methods before surgery have less anxiety, lower pain, less demand for painkillers, and shorter hospital stays [16].

VR technology creates an immersive and multisensory environment that provides patients with modified experiences of reality and creates, as a result, a sense of “presence” in the virtual world.

In virtual environments, the patients experience similar physiological symptoms and anxiety as they do in real-life situations, thereby facilitating the habituation process. Thus, patients undergo a process of systemic desensitization through gradual exposure to an anxiety- or fear-inducing stimulus, with the ultimate goal of minimizing the patients’ intense and adverse behavior towards the stimulus through a series of systematic steps. In addition, psychoeducation can be used as a method to change the patients’ cognition about the stimulus to reinforce treatment benefits of systemic desensitization [23].

Moreover, the patients are given control of immersive, complex, and dynamic 360° videos in the virtual space where they are sensorially inserted and will interact with and navigate within the VR environment. This initiative allows patients to align their expectations with reality, as VR helps to diminish the gap between imagination and the real world [24,25]. However, there is not much research that measures the effects of VR on preoperative anxiety and pain reduction in correlation to the postoperative period and reported outcomes. Therefore, we planned the present project to evaluate the effect of VR on preoperative anxiety and pain and its impact postoperatively.

VR can provide clinical value through its application to adults undergoing outpatient procedures by offering a novel non-pharmacologic means to address common patient needs. This innovation can lead to optimal patient care, reduced costs, fewer pharmacological side effects and enhanced staff and patient experiences during outpatient procedures.

## 2. Pathomechanism of Pain and Anxiety

There is a strong correlation between anxiety and pain. To comprehend the mechanistic beginnings for VR analgesia, the neurobiological connectivity of the brain’s cortices and neurochemistry have been investigated, including the attentional, emotional, and cognitive processes [26].

Various receptors and proteins have been revealed to mediate pain attributed to periphery, as well as at the spinal cord level, where the gate control of pain transmission is located [27].

Nonetheless, anxiety has been researched mainly within the context of neuropsychology. Recent research on neuroimaging has revealed the brain’s inner network and how it mediates interactions of pain and anxiety. Both healthy participants and patients with emotional symptoms had key cortical areas actively involved in both conditions, including the anterior cingulate cortex (ACC), insular cortex (IC), and amygdala. Moreover, integrative neurobiological tools have allowed the central synapses to be investigated, as well as their potential involvement in anxiety disorders and chronic pain [27,28,29,30,31].

The human brain’s response to painful stimuli has been captured in functional imaging studies, with activity in the insula, anterior cingulate gyrus, the thalamus, and the primary somatosensory cortex and the periaqueductal gray matter. These regions host a network of circuitry that is involved from the top-down, bottom-up, and intercortical processing of such painful stimuli, and are the origins of where pain is perceived.

Other studies have shown that pre-LTP in the ACC may affect spinal nociceptive transmission via top-down ACC spinal facilitatory systems. ACC neurons in deeper layers are said to project to spinal cord dorsal horn neurons, and stimulating the ACC can lead to nociceptive responses. Such effects are then mediated by descending serotonergic projections from the rostral ventromedial medulla (RVM) [32]. The activation of these dorsal neurons, in turn, triggers the incoming pain-related somatosensory cortices and results in pain being experienced in a similar way that certain regions of the body experience pain. Therefore, the ACC–spinal network could hold the mechanism which underlies anxiety-triggered body pain. Through the activation of the ACC top-down modulation of the dorsal horn, abnormal activities stemming from fear or anxiousness can trigger neuronal activity in the spinal cord while interacting with the everyday ongoing sensory inputs from the periphery [33,34]. This would be enough of a stimulus for the pain-related areas of the cortex to activate, such as somatosensory cortex, ACC, and insular cortex, leading to a sensation of pain. Some activities may further improve top-down modulation via positive feedback.

NMDA receptors (NMDARs) play a significant role in pain associated with nerve injuries and peripheral tissues. Persistent activation of NMDARs can lead to increased sensitivity and responsiveness oof spinal cord neurons to such inputs, contributing to the central sensitization element of chronic pain [35]. Furthermore, NMDA-receptor-mediated postsynaptic LTP and kainate-receptor-mediated presynaptic LTP in the ACC are crucial for the perception of chronic pain and anxiety induced by physical harm [36,37]. It remains unclear if these synaptic regulation mechanisms also occur in other pain- or anxiety-related regions, as further research to investigate the modulatory mechanisms of the 5-HT system, at both the synaptic and molecular levels, is required [38].

However, these regions exhibit varying degrees of responses to aversive to non-aversive stimuli and a wide spectrum of task-based attention, distraction or affective conditions.

The responses observed by some subsets of these regions have been linked with a greater intensity of sensations and pain, as well as the unpleasantness of pain, as determined through imaging studies with well-controlled psychophysical measures. Despite this, challenges involving the separation of brain regions that process sensory information from those mediating the affective responses is still a controversial issue an area. Thus far, functional imaging or other investigative methods have failed to produce an objective, direct correlation of the pain experience, and have not clarified exactly how the brain’s sensory and affective mechanisms interact with one another to perceive pain as a reaction to potentially aversive stimuli.

Virtual reality has been proven to lessen pain, an effect known as “VR analgesia”. The subjective assessments of pain reduction through VR have been supported by functional MRI (fMRI) data, revealing decreased brain activity across regions typically highly active when exposed to experimental thermal pain stimulation [31]. Hoffman’s research, however, primarily focused on whether VR gaming, in its entirety, significantly diminishes increasing brain activities in the classic areas where pain is intrinsically linked with noxious thermal stimuli [39,40]. Another study compared the effects of VR to those of opioids on brain activities linked to thermal pain stimulation, and discovered that opioids and VR noticeably lessened pain-related brain activity in the insula and thalamus, but not in other areas of the pain circuitry.

Conversely, other cognitive activities have also been seen to reduce brain activity within the classic pain-processing circuitry during experimental pain stimulation. Valet et al. proposed that the cingulo–frontal cortex may apply top-down influences on periaqueductal gray matter and the posterior thalamus to lessen pain through distractions [41]. The heightened activity in the cingulo–frontal cortex might be a result of the increased cognitive load of such tasks and not of the mechanism of attention distraction itself [42]. Essentially, attention does not inherently require task loading; rather, task loading requires attention. Further research is needed to better understand these issues and to enhance our comprehension of the cortical processes responsible for pain modulation.

Despite there being studies reporting lower pain levels and increased comfort levels using VR glasses [43,44], little research has been conducted that specifically examines the overall impacts of virtual reality on anxiety in the context of neurosurgical and spinal surgery patients [45]. Therefore, this study aims to assess the effects of virtual reality applications on pain severity, anxiety level, and patient satisfaction in patients who undergo spinal surgery.

To the best of the authors’ knowledge, there is no previous study in the literature examining the impacts of virtual reality interventions when applied to three contrasting groups before neurosurgical or varying levels or types of spinal surgeries surrounding pain, anxiety, and vital signs. This study is being conducted to verify the effects of virtual reality intervention prior to spinal surgery on pain, anxiety, and vital signs.

## 3. Objectives

The aim of this research study is to evaluate the impact of immersive VR exposure on preoperative anxiety and pain, and, additionally, to assess whether VR intervention would significantly alleviate pain and anxiety levels of patients undergoing complex spinal surgeries. We hypothesize that the application of preoperative VR-based intervention will alleviate anxiety and pain both pre- and postoperatively when it is used as treatment to complement standard care compared to standard routine care alone, and that it may lead to better outcomes and higher satisfaction in patients. The primary objective is to evaluate whether procedural/educational VR reduces preoperative anxiety compared with standard care. Secondary objectives include comparing relaxation/distraction VR versus standard care, and procedural/educational VR versus relaxation/distraction VR with respect to postoperative pain, analgesic use, and patient satisfaction.

## 4. Material and Methods

### 4.1. Trial (Study) Design

This is a single-center study prospective, randomized, placebo-controlled trial with three parallel groups of adult patients (18 to 70 years) who have degenerative spinal disorders, specifically those diagnosed with lumbar or cervical spondylosis who are scheduled to undergo spinal fusion, discectomy, decompression, or stabilization procedures. Relevant surgical variables, including cervical/lumbar/thoracic location, type of surgery, surgical approach, duration of surgery, and perioperative complications, will be prospectively recorded and considered in adjusted analyses. The study assesses the influence of a single preoperative VR (exposure) session and its effect on both preoperative and postoperative anxiety and pain, as well as reported functional outcomes in patients undergoing elective spine surgery.

### 4.2. Trial Protocol Registration

The study protocol adheres to the Standard Protocol Items: Recommendations for Interventional Trials (SPIRIT) guidelines. Ethical approval was granted by the Regional Committee for Medical Research Ethics at the Medical University of Lodz. The study is registered with ClinicalTrials.gov (NCT06917300; 7 April 2025), under the unique protocol ID RNN/02/24/KE, and with the Clinical Trial Department of the Medical University of Lodz.

### 4.3. Participant Sample and Sample Size

#### 4.3.1. Recruitment and Allocation

The sample size calculation is based on detecting a clinically meaningful reduction in preoperative anxiety measured by the State-Trait Anxiety Inventory–State (STAI-S) score, which is the primary endpoint. The trial was powered based on the primary endpoint of STAI-S change. Prior VR perioperative studies report a mean reduction of 8–10 points with a standard deviation of approximately 10 points. Using Rosner’s formula for three parallel study arms, with α = 0.05, power = 0.80, and a minimum clinically significant difference of 8 points, 42 patients per arm are required. To account for an anticipated 15% attrition rate, we will enroll 50 participants per arm, for a total sample size of 150 patients. Elective procedures are defined as those happening the day after the initial patient evaluation. All patients will be provided with written informed consent, and the trial procedures will be explained verbally and in writing to all patients prior to study enrolment.

#### 4.3.2. Study Group Selection

The following inclusion and exclusion criteria (Table 1) were systematically established in order to guide the recruitment process and determine eligibility for enrolment in this trial.

### 4.4. Randomization

Eligible patients will be randomly assigned to the VR group, the control group (standard preoperative care), and the VR relaxation/distraction group. Randomization will be performed using a computer-generated permuted block randomization sequence (block size = 6) created by a statistician not involved in patient recruitment or assessment. Allocation concealment will be ensured using sequentially numbered, opaque, sealed envelopes opened only after enrolment. Participants cannot be fully blinded to VR exposure; however, they will be informed that two different VR experiences are under investigation without emphasis on superiority. Outcome assessors and data analysts will remain blinded to treatment allocation. An active comparator VR relaxation/distraction group is included to minimize expectancy and attention-related bias.

After obtaining the patients’ consent and agreement to undergo surgery, the randomization and allocation sequence will be carried out blindly by an independent research assistant during the clinic visit. The researcher conducting interviews and collecting the primary and secondary outcome data will be blind to the group assignments.

To minimize selection bias, randomization will be performed by an investigator independent of patient recruitment and assessment, and outcome assessors and data analysts will remain blinded to group allocation. Participants will be informed that both interventions are active and under evaluation without emphasis on superiority. To reduce the risk of dropout, all participants will receive equal attention and standardized information regarding the importance of study participation. The VR intervention will be administered on the day prior to surgery in a quiet preoperative preparation room.

To prevent any setbacks, a limited number of people will be included to interact with the patients, and a reminder will be given to the patients not to discuss the trial procedure and allocations. In addition, the experiment room will be different from the regular patients’ rooms. In the experiment room, the patients will be exposed to VR content, with respective psychological and physiological assessments. After that, the patients will be instructed to go back to their hospital rooms.

### 4.5. Instruments and VR Utilization

The study affects all three groups, and the VR intervention includes only one session. All intervention sessions will be carried out individually. After these assessments, the patients will be oriented to the VR goggles and headset and given tutorials and instructions on how to use the device and how to remove it when they encounter potentially unwanted side effects such as dizziness, headache, nausea, or vomiting. In such cases, the procedure will be stopped and considered a withdrawal.

On the day before surgery, following baseline assessment and informed consent, the researcher will randomly allocate the patients to the VR group, control group, and VR relaxation/distraction group.

The study timeline will proceed as follows: enrollment and informed consent → baseline psychometric and pain assessments → randomization → VR or standard-care intervention on the day before surgery → immediate postintervention assessment → surgery → postoperative assessment within 24 h and analgesic assessment within 24–48 h postoperatively.

VR-related adverse effects, including dizziness, nausea, headache, cybersickness, and visual discomfort, will be prospectively recorded and analyzed descriptively throughout the intervention period.

### 4.6. VR Device

For the intervention group, the Oculus Meta Quest 2 Goggles device (Meta Platforms, Inc., Menlo Park, CA, USA) will be used. It is an advanced and immersive HMD with ultra-short-focus optical technology. The VR device comes with a precision controller that acts as a natural extension of one’s hands. Improved tracking capabilities mean that it can also be operated controller-free. These features are designed to effectively address blurring at the edges of the field of view to reduce dizziness and improve user comfort and immersion.

### 4.7. Intervention in Each Group

#### 4.7.1. VR Intervention Group

The day before the surgery, the VR procedure will be explained to the patients while they wait for surgery. The VR group will be exposed to a unified 10 min VR video via Meta Quest 2 Goggles after receiving routine standard care. A 360° video with an immersive audio–visual environment describing the preoperative and postoperative experience on the day of the surgery will be provided, by which the patients can get accustomed to the environment and procedures associated with the surgery. Background audio will be provided through headsets, and the video will be directed by a neurosurgeon filmed using a Max 360 action VR camera (GoPro, Inc., San Mateo, California, USA). In the video, actors, neurosurgeons, and nurses will re-enact a typical day for a mock patient undergoing surgery, plus routine postoperative care, including the postoperative immediate recovery room and rehabilitation care by neurosurgeons, psychologists, and physiotherapists in the ward. Patients are allowed to move around freely so that they can experience all aspects of the virtual space, and are encouraged to ask questions at the end.

#### 4.7.2. VR Relaxation/Distraction Intervention Group Patients

The VR relaxation/distraction intervention group will be provided with standard routine care followed by a VR intervention designed to facilitate relaxation and distraction. This intervention utilizes numerous highly immersive environments, each stimulating a distinct natural setting (i.e., natural landscapes, urban parks, tropical beaches and forests) with background music, delivered via a commercially available application (i.e., Nature Treks VR (Greensky Games, London, UK), accessed via the Meta Quest platform (Meta Platforms, Inc., Menlo Park, CA, USA)) through a head-mounted display (HMD) and headsets. Patients can navigate these environments using a handheld controller, engaging in context-specific activities such as walking along a beach or climbing a mountain. These interactions are accompanied by dynamic environmental changes, which enhance the sense of presence, facilitate immersive engagement, and contribute a vivid experience.

These interventions will be delivered and preoperative assessment will take place on the day before surgery or at the moment the patient is recruited. Additionally, the postoperative assessment will be conducted within 24 h after the operation. The rationale for comparing both interventions is to explore whether cognitive (information-based) or emotional (relaxation-based) mechanisms provide greater benefit in the perioperative context.

We aimed at assessing the value of educational and exposure to stress factors in comparison to the second (2nd) intervention, where we use various relaxing immersive and game-based destinations that patients have access to prior to their respective surgeries.

#### 4.7.3. Standard Care (Control) Group

In the standard group, patients will only receive standard care and be provided with routine descriptions of the preoperative experience, where a surgeon will explain to them what the preoperative experience will entail.

After VR intervention, psychometric assessments of anxiety, pain score, and satisfaction will be performed and evaluated shortly before the intended surgery. Subsequently, the patients will undergo their planned surgical procedure. Postoperative data on analgesic use and pain will also be collected in the recovery area.

#### 4.7.4. Clinical Outcome Variables and Assessment

The primary outcome is the change in STAI-S score from baseline to immediately after intervention. Secondary outcomes include postoperative pain intensity assessed using the Visual Analogue Scale (VAS), postoperative analgesic use, patient satisfaction, and VR-related adverse effects. These outcomes will be measured preoperatively on the day before surgery, before the VR content exposure, and after. Measurements will be made again postoperatively—within 24 h. Preoperative and postoperative STAI-S and VAS will be compared for data analysis.

The baseline assessment is conducted one day before surgery, prior to the VR intervention, to capture the patient’s preoperative state under conditions closest to the actual surgical context. We believe this timing best reflects the anxiety and pain levels that the VR intervention aims to reduce. The postoperative assessment within 24 h is intended to evaluate the immediate effect of the intervention on perioperative anxiety and pain, which is the primary focus of our study. We acknowledge that longer-term outcomes may differ, but these are beyond the scope of the current trial, which specifically targets the short-term perioperative period (see Table 2).

The secondary outcome will be the patients’ quality of life and satisfaction (EVAN-G) of the VR intervention, time spent thinking about pain assessed, and postoperative analgesics measured from patient treatment charts within 24 and 48 h postoperatively and used for data analysis, as the level of satisfaction and thinking time spent on pain can contribute to an increased perception of pain.

### 4.8. Data Collection

#### Baseline Value Measurement

On the day before surgery, after informed consent is obtained, evaluations, which include sociodemographic data and the psychometric questionnaires (STAI-S, PSS-10, BDI-II), pain score (VAS), and satisfaction (EVAN-G), will be collected by the researcher before the VR experience (see Table 3).

Perioperative anesthesia and analgesia management will follow standardized institutional protocols whenever feasible. Variables including anesthetic technique, intraoperative opioid administration, postoperative opioid consumption, rescue analgesic use, surgery duration, and perioperative complications will be prospectively recorded and considered in adjusted secondary analyses.

### 4.9. Statistical Analysis

The statistical analysis will be performed under the mentorship of a biostatistician experienced in neurosurgical research. The nominal data will be presented as *n* (% of the total). The dependencies between them will be assessed using the test chosen based on the size of the smallest sample. All analyses will follow an intention-to-treat approach. The primary endpoint (change in STAI-S score from baseline to immediately postintervention) will be analyzed using one-way ANCOVA adjusted for baseline STAI-S. Pairwise comparisons will be Bonferroni-corrected. Secondary outcomes will be analyzed as follows: VAS pain at 24 h (ANCOVA adjusted for opioid dose), total opioid consumption (ANOVA or Kruskal–Wallis depending on distribution), patient satisfaction (Kruskal–Wallis), and rescue analgesic use (χ^2^ test). Longitudinal measures (baseline → post-VR → postoperative) will be evaluated with a linear mixed-effects model including group, time, and group × time interaction. Normality will be tested using the Shapiro–Wilk test. Missing data will be assessed for pattern and mechanism. Multiple imputation (m = 10) will be used if appropriate. Longitudinal analyses will primarily rely on linear mixed-effects models, which are robust to missing observations under missing-at-random assumptions. The primary comparison will evaluate procedural/educational VR versus standard care with respect to STAI-S score change. Secondary comparisons will include relaxation/distraction VR versus standard care and procedural/educational VR versus relaxation/distraction VR. Postoperative pain analyses will additionally be adjusted for perioperative opioid consumption and surgery duration when appropriate. Statistical analysis will be performed using STATISTICA 13.1 (TIBCO, Palo Alto, Santa Clara, CA, USA) and two-sided α = 0.05.

## 5. Discussion

It is our understanding that there have not been any previous clinical trials or case studies conducted to determine the effects of VR-based interventions on preoperative pain and anxiety among neurosurgical patients. Preoperative anxiety has detrimental consequences as it raises postoperative complications, behavioral disorders, and can be disturbing emotionally.

Preoperative anxiety may occur due to multiple factors, such as former negative experiences, being exposed to terrifying stereotypes of doctors in the media, or even triggered by friends, family members, and colleagues [46]. Some common fears linked to preoperative anxiety are the fear of pain itself, blood injuries, and hospital settings. Milgrom et al. revealed four groups of anxiety-driven patients based on the origins/sources of the patients’ fear. Some were anxious due to specific surgical stimuli, distrust of hospital workers, general dental anxiety, and fear of the unknown. It was discovered that more invasive types of stimuli were more anxiety-provoking; however, stimuli linked to clinic offices, team members, and/or the equipment being used were the least fear-provoking. It was revealed that most of the patients felt anxious during their first clinical visit. Their main concerns were how anesthesia would be administrated, extraction, feelings of numbness, and the root canal treatment itself [47]. Recent research has connected anxiety with pain as it increases sympathetic activity, thereby releasing endogenous adrenaline and increased pain through nociceptors [48].

Chaves et al. surmised that there were no major differences between blood pressure (BP) and anxiety for either gender [49]. Conceição et al. found no statistically significant differences in heart rate (HR) and BP of anxious and non-anxious patients [50].

The purpose of this research is to further explore the beneficial impacts of VR on alleviating preoperative anxiety and levels of pain by examining three study groups. Prior reports have indicated that VR intervention can lessen the anxiety experienced by patients before surgery; however, other studies have found no such benefits among adult patients. Additionally, digital distraction techniques have been proven to lower stress and pain levels during interventions and painful procedures.

Consequently, distraction techniques and non-pharmacological methods are paramount for simple, easily implementable interventions that can reduce anxiety and disruptive behaviors among patients. VR is a significant advancement in modern technology to simulate reality, transforming the philosophical concept of the virtual world into a practical experience [51]. It is a new medium that surpasses mere illusion and can produce real-world results due to its unlimited potential for large-scale applications in not only training and treatment, but also therapy. VR not only helps to visualize events but also to experience psychological conditions and personalized perceptions, fostering a sense of ownership, presence, and immersion [52,53]. It can also influence physical sensations during interventions, including stress and anxiety reduction and pain management, while inducing the necessary emotions [54,55].

Virtual reality headsets and related devices used for patients about to undergo surgical intervention, such as spinal stabilization, laminectomy, discectomy, and stimulation, can help to reduce acute pain and anxiety, leading to higher levels of patient satisfaction. Various other non-pharmacological approaches have been employed to lessen anxiety, stress, and pain, such as gamification, watching cartoons, and art music therapy. Gamification and preoperative multimedia education are two other resources that may be effective throughout the preparation phases before surgery and in postoperative care. Studies have outlined that children who undergo an education-based multimedia intervention are less anxious and stressed about surgery and other hospital procedures.

In spite of the significant pain and anxiety experienced by many patients prior to undergoing a surgical procedure, non-pharmacological approaches are still seldomly implemented as a means or tool for reducing anxiety or pain-related signals. VR video intervention is able to lessen preoperative anxiety while providing the chance to teach and familiarize patients in the hospital environment, be it for an inpatient or even at home, before attending for surgery. VR can also improve the physical, mental, and emotional state of each patient.

Sato et al. investigated the use of VR for the treatment of complex regional pain syndrome in patients and was able to halve the pain intensity scores [56]. This correlates with our study, wherein a significant reduction in the postoperative pain perception of the patient was observed. Sarig-Bahat et al. used VR to treat 67 patients with chronic neck pain and discovered that VR usage for a single session raised the cervical range of motion and lessened neck pain [57]. Hoffman et al. used VR for physical therapy sessions for burn victims and discovered that the perception of pain for his patients was considerably less when using VR, which confirms the results of our study [13]. In a simulation study conducted by Tanja-Dijkstra et al., it was revealed that the use of VR as a means of distraction can potentially influence a patient’s perception of an anxiety-inducing procedure [58]. Jeffery I Gold et al. concluded that VR usage lowers anxiety by preventing vivid memories from being formed [59]. Conversely, although the pros of VR intervention seem significant, not much is known surrounding the longer-term effects, such as after surgery, during hospitalization or after discharge.

This study delved into the effectiveness of distractions and immersion-based virtual reality in the context of preoperative anxiety among patients undergoing spinal surgery. Using virtual reality to lessen or distract attention away from the hospital environment seems to allow patients to tolerate preoperative anxiety. The VR exposes patients to worrying and unfamiliar situations in a safe environment before the real procedure takes place. This technique/intervention can result in a sensation of familiarity and, thus, result in reduced anxiety.

## 6. Limitations

The limitations of this study are a lack of consideration of physiological signs and hemodynamic parameters (BP, HR) of patients before surgery, and a lack of homogenization concerning the type of surgery. Moreover, the patients in this study varied in age, previous experiences, education levels, procedure types, and hospital settings. This means that the current study should be carefully conducted, with the findings cautiously generalized as if all patients were the same age. Therefore, distraction-based intervention needs to be further researched and optimized for surgical pathways, and enhancements concerning preoperative and postoperative clinical settings for the patients are needed. Additional limitations include potential expectancy effects, incomplete participant blinding, possible attention/time-contact imbalance between intervention groups and standard care, heterogeneity in surgical procedures, and the relatively short-term postoperative follow-up period.

## Figures and Tables

**Table 1:** Eligibility Criteria for Study Enrollment.

Inclusion Criteria	Exclusion Criteria
Adult patients between 18–70 years old Patient’s admission prior to surgery day (at least 1 day) Patients’ first surgery with no prior surgical history cervical vs. lumbar vs. thoracic) Type of spine surgery (e.g., cervical vs. lumbar vs. thoracic) Surgical approach (e.g., minimally invasive vs. open) Anesthetic technique (e.g., general) Expected hospital stay—2 days Capable of providing informed consent and participate in the study follow-up questionnaire Patients speak English	Congestive heart failure, hypertension and anti-hypertensive medications Adrenal insufficiency Cognitive impairment (evaluated by MMSE, MoCA) Cerebrovascular diseases, ophthalmological diseases (strabismus, diplopia, retina degeneration, glaucoma), neurological conditions (epilepsy or seizure, dementia, vertigo, dizziness, motion sickness)—evaluated by: history taking, examination, vision test (Ishihara test) Auditory impairment Psychiatric illness (schizophrenia, bipolar disorder, psychotic depression)—(evaluated by MINI questionnaire) Patients with neuropathic pain Use of sedative drugs, anti-depressants, anxiolytics, and anti-epileptic drugs Claustrophobia GCS < 15

**Legend:** MMSE, Mini-Mental State Examination; MoCA, Montreal Cognitive Assessment; MINI, The Mini International Neuropsychiatric Interview; GCS, Glasgow Coma Scale.

**Table 2:** Primary and secondary outcome assessment.

Timepoint	Assessment	Measures
Day before surgery (pre-VR)	Baseline	STAI-S, VAS pain
Day before surgery (post-VR)	Immediately after VR intervention	STAI-S, VAS pain, patient experience satisfaction
Within 24 h after surgery	Postoperative	VAS pain, analgesic use

**Table 3:** Preoperative and postoperative psychometric scales.

Baseline Values
Patients’ demographics: age, sex, education, medical history (comorbidities), medication, surgery type, duration, ASA status Anxiety (STAI-S) Stress (PSS-10) Depression (BDI-II) Pain score (VAS) Satisfaction (EVAN-G) Cognitive (ACE-III) Personality test (PID-5) Postoperative analgesia (Medical charts and records)

**Legend:** ASA, American Society of Anesthesiologists; STAI-S: State-Trait Anxiety Inventory Scale; PSS-10, Perceived Stress Scale; BDI-II, Beck Depression Inventory; VAS, Visual Analogue Scale; EVAN-G, Evaluation du Vecu de l’Anesthesie Generale; ACE-III, Addenbrooke’s Cognitive Examination; PID-5, Personality Inventory for DSM-5.

## Data Availability

No new data were created or analyzed in this study.

## References

[1] Woldegerima Y.B., Fitwi G.L., Yimer H.T., Hailekiros A.G. (2018). Prevalence and factors associated with preoperative anxiety among elective surgical patients at University of Gondar Hospital. Gondar, Northwest Ethiopia, 2017. A cross-sectional study. Int. J. Surg. Open.

[2] Corman H.H., Hornick E.J., Kritchman M., Terestman N. (1958). Emotional Reactions of Surgical Patients to Hospitalization, Anesthesia and Surgery. Am. J. Surg..

[3] Wallace L.M. (1984). Psychological preparation as a method of reducing the stress of surgery. J. Hum. Stress.

[4] Shevde K., Panagopoulos G. (1991). A Survey of 800 Patients’ Knowledge, Attitudes, and Concerns Regarding Anesthesia. Anesth. Analg..

[5] Hicks J.A., Jenkins J.G. (1988). The measurement of preoperative anxiety. J. R. Soc. Med..

[6] Uğraş G.A., Yıldırım G., Yüksel S., Öztürkçü Y., Kuzdere M., Öztekin S.D. (2018). The effect of different types of music on patients’ preoperative anxiety: A randomized controlled trial. Complement. Ther. Clin. Pract..

[7] Matthias A.T., Samarasekera D.N. (2012). Preoperative anxiety in surgical patients—Experience of a single unit. Acta Anaesthesiol. Taiwanica.

[8] Namazi M., Amir Ali Akbari S., Mojab F., Talebi A., Alavi Majd H., Jannesari S. (2014). Aromatherapy with citrus aurantium oil and anxiety during the first stage of labor. Iran. Red Crescent Med. J..

[9] Dehghan F., Jalali R., Bashiri H. (2019). The effect of virtual reality technology on preoperative anxiety in children: A Solomon four-group randomized clinical trial. Perioper. Med..

[10] Butler R.K., Finn D.P. (2009). Stress-induced analgesia. Prog. Neurobiol..

[11] Liu Y., Wang R., Zhang Y., Feng L., Huang W. (2023). Virtual reality psychological intervention helps reduce preoperative anxiety in patients undergoing carotid artery stenting: A single-blind randomized controlled trial. Front. Psychol..

[12] Friedrich S., Reis S., Meybohm P., Kranke P. (2022). Preoperative anxiety. Curr. Opin. Anaesthesiol..

[13] Hoffman H.G., Patterson D.R., Carrougher G.J., Sharar S.R. (2001). Effectiveness of virtual reality-based pain control with multiple treatments. Clin. J. Pain.

[14] Fayazi S., Babashahi M., Rezaei M. (2011). The effect of inhalation aromatherapy on anxiety level of the patients in preoperative period. Iran. J. Nurs. Midwifery Res..

[15] Cho M.-K., Choi M.-Y. (2021). Effect of distraction intervention for needle-related pain and distress in children: A systematic review and meta-analysis. Int. J. Environ. Res. Public Health.

[16] Bekelis K., Calnan D., Simmons N., MacKenzie T.A., Kakoulides G. (2017). Effect of an Immersive Preoperative Virtual Reality Experience on Patient Reported Outcomes: A Randomized Controlled Trial. Ann. Surg..

[17] Guo C., Deng H., Yang J. (2015). Effect of virtual reality distraction on pain among patients with hand injury undergoing dressing change. J. Clin. Nurs..

[18] Schwartz H.J., Fagan S., Craft-Coffman B., Truelove C.A., Mullins R.F. (2020). 124 Virtual Reality for Reducing Pain and Perioperative Anxiety in Pediatric Burn Patients. J. Burn Care Res..

[19] Sweta V.R., Abhinav R.P., Ramesh A. (2019). Role of Virtual Reality in Pain Perception of Patients Following the Administration of Local Anesthesia. Ann. Maxillofac. Surg..

[20] Moon J.Y., Shin J., Chung J., Ji S.-H., Ro S., Kim W.H. (2018). Virtual Reality Distraction during Endoscopic Urologic Surgery under Spinal Anesthesia: A Randomized Controlled Trial. J. Clin. Med..

[21] Indovina P., Barone D., Gallo L., Chirico A., De Pietro G., Giordano A. (2018). Virtual Reality as a Distraction Intervention to Relieve Pain and Distress During Medical Procedures: A Comprehensive Literature Review. Clin. J. Pain.

[22] Gupta A., Scott K., Dukewich M. (2018). Innovative Technology Using Virtual Reality in the Treatment of Pain: Does It Reduce Pain via Distraction, or Is There More to It?. Pain Med..

[23] Oing T., Prescott J. (2018). Implementations of Virtual Reality for Anxiety-Related Disorders: Systematic Review. JMIR Serious Games.

[24] De Carvalho M.R., Freire R.C., Nardi A.E. (2010). Virtual reality as a mechanism for exposure therapy. World J. Biol. Psychiatry.

[25] Wilson P.N., Foreman N., Tlauka M. (1996). Transfer of spatial information from a virtual to a real environment in physically disabled children. Disabil. Rehabil..

[26] Li A., Montaño Z., Chen V.J., Gold J.I. (2011). Virtual reality and pain management: Current trends and future directions. Pain Manag..

[27] Rutter C.E., Dahlquist L.M., Weiss K.E. (2009). Sustained Efficacy of Virtual Reality Distraction. J. Pain Off. J. Am. Pain Soc..

[28] Hoffman H.G., Richards T.L., Van Oostrom T., Coda B.A., Jensen M.P., Blough D.K., Sharar S.R. (2007). The analgesic effects of opioids and immersive virtual reality distraction: Evidence from subjective and functional brain imaging assessments. Anesth. Analg..

[29] Hoffman H.G., Richards T.L., Coda B., Bills A.R., Blough D., Richards A.L., Sharar S.R. (2004). Modulation of thermal pain-related brain activity with virtual reality: Evidence from fMRI. NeuroReport.

[30] Hoffman H.G., Richards T.L., Bills A.R., Van Oostrom T., Magula J., Seibel E.J., Sharar S.R. (2006). Using FMRI to study the neural correlates of virtual reality analgesia. CNS Spectr..

[31] Bantick S.J., Wise R.G., Ploghaus A., Clare S., Smith S.M., Tracey I. (2002). Imaging how attention modulates pain in humans using functional MRI. Brain.

[32] Zhuo M. (2016). Neural Mechanisms Underlying Anxiety–Chronic Pain Interactions. Trends Neurosci..

[33] Johansen J.P., Fields H.L. (2004). Glutamatergic activation of anterior cingulate cortex produces an aversive teaching signal. Nat. Neurosci..

[34] Chen T., Taniguchi W., Chen Q.-Y., Tozaki-Saitoh H., Song Q., Liu R.-H., Koga K., Matsuda T., Kaito-Sugimura Y., Wang J. (2018). Top-down descending facilitation of spinal sensory excitatory transmission from the anterior cingulate cortex. Nat. Commun..

[35] Bird G.C., Lash L.L., Han J.S., Zou X., Willis W.D., Neugebauer V. (2005). Protein kinase A-dependent enhanced NMDA receptor function in pain-related synaptic plasticity in rat amygdala neurones. J. Physiol..

[36] Koga K., Descalzi G., Chen T., Ko H.G., Lu J., Li S., Son J., Kim T.H., Kwak C., Huganir R.L. (2015). Coexistence of two forms of LTP in ACC provides a synaptic mechanism for the interactions between anxiety and chronic pain. Neuron.

[37] Bliss T.V.P., Collingridge G.L., Kaang B.-K., Zhuo M. (2016). Synaptic plasticity in the anterior cingulate cortex in acute and chronic pain. Nat. Rev. Neurosci..

[38] Villas-Boas G.R., Lavorato S.N., Paes M.M., de Carvalho P.M.G., Rescia V.C., Cunha M.S., de Magalhães-Filho M.F., Ponsoni L.F., de Carvalho A.A.V., de Lacerda R.B. (2021). Modulation of the Serotonergic Receptosome in the Treatment of Anxiety and Depression: A Narrative Review of the Experimental Evidence. Pharmaceuticals.

[39] Wise R.G., Rogers R., Painter D., Bantick S., Ploghaus A., Williams P., Rapeport G., Tracey I. (2002). Combining fMRI with a pharmacokinetic model to determine which brain areas activated by painful stimulation are specifically modulated by remifentanil. Neuroimage.

[40] Porro C.A. (2003). Functional imaging and pain: Behavior, perception, and modulation. Neuroscientist.

[41] Valet M., Sprenger T., Boecker H., Willoch F., Rummeny E., Conrad B., Erhard P., Tolle T.R. (2004). Distraction modulates connectivity of the cingulo-frontal cortex and the midbrain during pain—An fMRI analysis. Pain.

[42] Ong W.-Y., Stohler C.S., Herr D.R. (2018). Role of the Prefrontal Cortex in Pain Processing. Mol. Neurobiol..

[43] Nilsson S., Finnström B., Kokinsky E., Enskär K. (2009). The use of Virtual Reality for needle-related procedural pain and distress in children and adolescents in a paediatric oncology unit. Eur. J. Oncol. Nurs..

[44] Scapin S., Echevarría-Guanilo M.E., Boeira Fuculo Junior P.R., Gonçalves N., Rocha P.K., Coimbra R. (2018). Virtual Reality in the treatment of burn patients: A systematic review. Burns.

[45] Keshvari M., Yeganeh M.R., Paryad E., Roushan Z.A., Pouralizadeh M. (2021). The effect of virtual reality distraction on reducing patients’ anxiety before coronary angiography: A randomized clinical trial study. Egypt. Heart J..

[46] Appukuttan D.P. (2016). Strategies to manage patients with dental anxiety and dental phobia: Literature review. Clin. Cosmet. Investig. Dent..

[47] Milgrom P. (1985). Treating Fearful Dental Patients: A Patient Management Handbook.

[48] Janssen S.A., Arntz A., Bouts S. (1998). Anxiety and pain: Epinephrine-induced hyperalgesia and attentional influences. Pain.

[49] Chaves E.C., Cade N.V. (2004). Anxiety effects on blood pressure of women with hypertension. Rev. Lat. Am. Enferm..

[50] Goulart J.C.F., Pinheiro M.D., Rodrigues R.V., dos Santos F.D.S.A., Martins A.T., Scannavino F.L.F. (2012). Influence of anxiety on blood pressure and heart rate during dental treatment. Rev. Odonto Cienc..

[51] Georgiev D.D., Georgieva I., Gong Z., Nanjappan V., Georgiev G.V. (2021). Virtual Reality for Neurorehabilitation and Cognitive Enhancement. Brain Sci..

[52] Schutte N.S. (2020). The Impact of Virtual Reality on Curiosity and Other Positive Characteristics. Int. J. Hum.-Comput. Interact..

[53] Martens M.A.G., Antley A., Freeman D., Slater M., Harrison P.J., Tunbridge E.M. (2019). It feels real: Physiological responses to a stressful virtual reality environment and its impact on working memory. J. Psychopharmacol..

[54] Jones T., Skadberg R., Moore T. (2018). A Pilot Study of the Impact of Repeated Sessions of Virtual Reality on Chronic Neuropathic Pain. Int. J. Virtual Real..

[55] Ahmadpour N., Weatherall A.D., Menezes M., Yoo S., Hong H., Wong G. (2020). Synthesizing multiple stakeholder perspectives on using virtual reality to improve the periprocedural experience in children and adolescents: Survey study. J. Med. Internet Res..

[56] Sato K., Fukumori S., Matsusaki T., Maruo T., Ishikawa S., Nishie H., Takata K., Mizuhara H., Mizobuchi S., Nakatsuka H. (2010). Nonimmersive virtual reality mirror visual feedback therapy and its application for the treatment of complex regional pain syndrome: An open-label pilot study. Pain Med..

[57] Sarig-Bahat H., Weiss P.L., Laufer Y. (2010). Neck pain assessment in a virtual environment. Spine.

[58] Tanja-Dijkstra K., Pahl S., White M.P., Andrade J., Qian C., Bruce M., May J., Moles D.R. (2014). Improving Dental Experiences by Using Virtual Reality Distraction: A Simulation Study. PLoS ONE.

[59] Gold J.I., Belmont K.A., Thomas D.A. (2007). The neurobiology of virtual reality pain attenuation. Cyberpsychol. Behav..

